# Removal of all ovarian tissue versus conserving ovarian tissue at time of hysterectomy in premenopausal patients with benign disease: study using routine data and data linkage

**DOI:** 10.1136/bmj.j372

**Published:** 2017-02-06

**Authors:** Jemma Mytton, Felicity Evison, Peter J Chilton, Richard J Lilford

**Affiliations:** 1 University Hospitals Birmingham NHS Foundation Trust, Birmingham B15 1JD, UK; 2 Warwick Business School, University of Warwick, Coventry CV4 7AL, UK; 3 Warwick Medical School, University of Warwick, Coventry CV4 7AL, UK

## Abstract

**Objective** To conduct a nationwide study of associations between removal of all ovarian tissue versus conservation of at least one ovary at the time of hysterectomy and important health outcomes (ischaemic heart disease, cancer, and all cause mortality).

**Study design and setting** Retrospective analysis of the English Hospital Episode Statistics database linked to national registers of deprivation indices and of deaths.

**Participants** 113 679 patients aged 35-45 who had had a hysterectomy for benign conditions between April 2004 and March 2014.

**Exposures** Bilateral ovarian removal versus no removal or unilateral ovarian removal (ovarian conservation).

**Main outcome measures** Hospital admissions for ischaemic heart disease, cancer, or attempted suicide; deaths, overall and from heart disease, cancer, or suicide. Statistical adjustments were made using Cox regression and propensity score matching for potential confounders.

**Results** A third of patients had bilateral ovarian removal. Patients in the ovarian conservation group were less likely to be admitted for ischaemic heart disease after hysterectomy than were those in the bilateral removal group (adjusted hazard ratio 0.85, 95% confidence interval 0.77 to 0.93; P=0.001). They were also less likely to have a cancer related post-hysterectomy admission (adjusted hazard ratio 0.83, 0.78 to 0.89; P<0.001). A significant difference in all cause mortality was also seen: 0.60% (456/76 581) of patients with ovarian conservation compared with 1.01% (376/37 098) of patients with bilateral removal. Again, this difference in favour of ovarian conservation was significant (adjusted hazard ratio 0.64, 0.55 to 0.73; P<0.001). Fewer deaths related specifically to heart disease (adjusted hazard ratio 0.50, 0.28 to 0.90; P=0.02) and to cancer (0.54, 0.45 to 0.65; P<0.001) occurred in the ovarian conservation group than in the bilateral removal group. No significant difference between groups was found relating to suicide (attempted or completed). The results after propensity score matching were essentially unchanged.

**Conclusion** Patients who had ovarian conservation had a significantly lower hazard of all cause mortality compared with those who had bilateral ovarian removal and also had lower death rates from ischaemic heart disease and cancer. Consistent with this observation, admissions to hospital for both ischaemic heart disease and cancer were also lower in the ovarian conservation group than in the bilateral removal group. Although removal of both ovaries protects against subsequent development of ovarian cancer, premenopausal women should be advised that this benefit comes at the cost of an increased risk of cardiovascular disease and of other (more prevalent) cancers and higher overall mortality.

## Introduction

Strong arguments exist to remove both ovaries at the time of hysterectomy in women who have reproductive cancers or who have a high risk of developing cancers. Cancers of the breast or endometrium are often stimulated by ovarian hormones, and cancer in one ovary often spreads to the other. However, many premenopausal women who have no such specific indication nevertheless have both healthy ovaries removed at the time of hysterectomy as a prophylactic measure to forestall the later development of ovarian cancer. Empirical investigations have confirmed the intuitive conclusion that such a measure protects against the risk of ovarian cancer—the hazard ratio over 28 years’ follow-up was 0.06 (95% confidence interval 0.02 to 0.21) in the large Nurses’ Health Study.^1^ The combination of biological plausibility and the massive “effect size” make a compelling case that women can be advised that their risk of ovarian cancer is greatly reduced by oophorectomy. However, the lifetime risk of developing ovarian cancer is one in 52 in the UK,^2^ and the removal of a metabolically active organ such as the ovary may have harmful effects in the long term. If so, these long term disadvantages (combined with the unpleasant shorter term effects of acute oestrogen deprivation) must be offset against the benefit conferred by protection from ovarian cancer. This possibility has been investigated in several studies, the largest of which is the Nurses’ Health Study, in which a cohort of 30 117 participants had a hysterectomy for benign disease.^1^ ^3^ All cause mortality, coronary heart disease mortality, and deaths from all cancers were significantly decreased when ovarian tissue was conserved compared with when both ovaries were removed. Several other cohort studies have been published. These are small compared with the Nurses’ Health Study, and they classify outcomes in different ways—for instance, combining heart attack, heart failure, and stroke. They confirm an association between removal of all ovarian tissue and an increased risk of cardiovascular disease, all cause mortality, or both.^4^ ^5^ ^6^ ^7^

We used a national database of hospital admissions and linked it to the national register of deaths to conduct a nationwide study of the putative associations between removal of all ovarian tissue and important health outcomes. Our intention was to conduct an even larger study than the Nurses’ Health Study, to do so on a countrywide basis rather than in a sample, and to examine associations between operation type and subsequent hospital admissions, as well as mortality.

## Methods

### Framing the question

This study is based on linkage of the English Hospital Episode Statistics (HES) and the national registration of deaths (Office for National Statistics (ONS)). The Health and Social Care Information Centre produces a yearly report on the quality of HES data. The accuracy of recording of primary diagnosis and primary procedures has been consistent between 2010/11 and 2012/13 (99.3% and 99.9%, respectively).^8^ Any “database study” is constrained by information included in the databases and how it is coded.

We took several a priori decisions. Firstly, we included women between the age of 35 and 45. The upper limit was designed to ensure that the great majority of cases would be premenopausal, so a strong argument existed to retain ovarian tissue for its putative beneficial endocrine effects. The lower limit was selected so that included women were typical of the majority in whom this decision would be encountered in clinical practice, given that hysterectomy is relatively uncommon under the age of 35. Secondly, we excluded cases with a history of reproductive cancer, including cancer of the breast. This conforms to the Nurses’ Health Study protocol and is salient to the clinical question concerning ovary removal in the absence of specific risk factors. Thirdly, we compared cases in which all ovarian tissue was removed with those with conservation of some ovarian tissue. Again, this is consistent with the primary analysis in the Nurses’ Health Study (and other studies) and with the clinical question of greatest relevance, as the decision to remove some ovarian tissue is typically dictated by incidental pathology (for example, discovery of a dermoid cyst in one ovary). Fourthly, outcomes included all cause mortality, mortality resulting from ischaemic heart disease and hospital admission for ischaemic heart disease, cancer (all cancers, ovarian cancer, breast cancer), and suicide. We then interrogated the database to select the intervention codes that would enable us to compare outcome rates by intervention type.

### Data selection

#### Selection of patients and generation of comparison groups

We collected data from the HES database on all patients aged between 35 and 45 who had a hysterectomy between 1 April 2004 and 31 March 2014 (corresponding to the end of the NHS “year”). We hold data from April 2001 and extracted data from 2004 onwards to allow for a minimum of three years of data to be accrued before the hysterectomy. The HES database contains information on all NHS funded admissions to hospitals in England. All admissions are given ICD-10 (international classification of disease, 10th revision) diagnosis codes and OPCS-4 (Office of Population Censuses and Surveys classification of interventions and procedures) procedure codes. The HES database is linked to the Indices of Multiple Deprivation database, and we were thus able to obtain the socioeconomic status of patients.^9^

We identified hysterectomies by OPCS codes (Q074, Q078, Q079, Q088, and Q089) and then categorised them into a bilateral removal group (bilateral ovary removal (Q221, Q223) or previous ovary removal followed by unilateral ovary removal at time of hysterectomy (Q231, Q232, Q235, Q236)) and an ovarian conservation group (no or unilateral ovary removal (as above)). We excluded patients who had a diagnosis of reproductive cancer (C51-C57) or breast cancer (C50) during a previous admission, along with those who had an ICD-10 code indicating that they had given a personal history of a reproductive cancer (Z854). We encountered a small proportion of women with codes indicating that peri-uterine tissue had been removed as part of the hysterectomy (Q071, Q073, Q073, Q081, Q082, Q083). Although we suspect that these are coding errors, removal of such tissue could signify existence or strong suspicion of cancer of the uterus, and we therefore excluded these cases. Lastly, we excluded hysterectomies conducted during an emergency admission, as this would lie outside the typical scenario in which the decision about ovary removal is made.

#### Covariates

We selected covariates recorded in HES on the basis of their known association with outcomes. We categorised patients’ ethnicity as “white,” “mixed,” “Asian or Asian British,” “black or black British,” “any other ethnic group,” or “unknown.” We scored comorbidity by using the Charlson comorbidity index. This score is derived from the sum of weighted scores of 17 medical conditions coded as comorbidities in the HES database. We treated the score as categorical and split it into groups of “0,” “less than 5,” “5 to 10,” “11 to 15,” and “more than 15.” A deprivation score for patients based on income, employment, health, education, training and skills, barriers to housing and services, crime, and living environment came from the Indices of Multiple Deprivation database. We used these scores to produce fifths, with fifth 1 being the most deprived and 5 being the least deprived. Other variables for risk adjustment/matching were age and number of previous admissions.

#### Outcomes

We recorded the following outcomes. (1) Emergency readmission rates calculated within both 30 days and 90 days of the index admission. Reason for admission was given by primary ICD-10 diagnosis codes. (2) Admissions for a myocardial infarction or other forms of ischaemic heart disease (I20-I25) (referred to collectively as ischaemic heart disease). We calculated the time between the index admission and first admission for ischaemic heart disease. (3) Admissions for subsequent cancers (overall and by organ of origin). (4) Admissions coded as attempted suicide (X60-X64). (5) Information on mortality, time to death, and cause of death, gathered using HES linked to the ONS mortality files. We classified cause of death as “heart disease,” cancer (overall and by organ of origin), and suicide.

### Statistical analysis

We tested all variables in a univariate analysis to examine whether an association existed between the type of ovary removal (ovarian conservation group versus bilateral removal group) and the probability of readmission to hospital either as an emergency within 90 days or due to ischaemic heart disease, cancer, or suicide. We included all the significant variables (P<0.05) in a multivariate Cox regression, which adjusted for age group, deprivation, removal type, and Charlson comorbidity score, as well as number of admissions before the hysterectomy. We produced Kaplan-Meier curves to analyse survival and fitted two separate Cox regression models, which included the significant variables and estimated the hazard of death and an ischaemic heart disease event or other event occurring. Proportional hazard assumptions were checked and satisfied using log-log plots.

We then created a matched dataset to test for a difference between women who had a bilateral removal and those who had one or no ovaries removed. We did this by generating a propensity score using the fitted values of a backwards step logistic model with the binary variable being ovarian conservation or bilateral removal. The two groups were matched on the above variables and also on the hospital in which the operation was performed to control for hospital level effects. We used the “greedy match” macro in SAS to match on a one to one ratio.

### Patient involvement

No patients were involved in setting the research question or the outcome measures, nor were they involved in developing plans for recruitment, design, or implementation of the study. No patients were asked to advise on interpretation or writing up of results. There are no plans to disseminate the results of the research to study participants or the relevant patient community.

## Results

### Patients

Between 1 April 2004 and 31 March 2014, 126 005 patients in the age range of 35-45 had a hysterectomy. Of these, we excluded 250 because sex or age was not recorded or their recorded residence was outside of England (these data are used, along with NHS number, to generate codes for follow-up). We also excluded patients if reproductive organ related cancer or breast cancer had been diagnosed at the time of or before their hysterectomy (4589). Other reasons for exclusion were hysterectomies with removal of peri-uterine tissue (2460), a personal history of reproductive cancer (3128), or emergency hysterectomy (2099). This left a study cohort of 113 679 (some women had more than one reason for exclusion), which we split into two groups for comparison—women with at least one ovary remaining (ovarian conservation: 76 581 (67.4%) patients) and those with no ovaries remaining (bilateral removal: 37 098 (32.6%) patients). The mean length of follow-up was 6.2 (SD 2.84) years.

Of the 113 679 patients in the study, 83 423 had an abdominal operation (Q074, Q078, or Q079), of whom 33 414 (40.1%) had bilateral ovarian removal. The other 30 256 had vaginal surgery (Q089 or Q088), of whom 3684 (12.2%) had bilateral ovarian removal. Table 1 gives further details.

**Table 1 tbl1:** Breakdown of demographics and clinical features of cohort of hysterectomy patients included in study. Values are numbers (percentages) unless stated otherwise

	Ovarian conservation group (n=76 581)	Bilateral removal group (n=37 098)	P value
**Region of residence**			
North East	4348 (5.7)	2235 (6.0)	<0.001
North West	11 746 (15.3)	6312 (17.0)	
Yorkshire and Humber	8801 (11.5)	3200 (8.6)	
East Midlands	7196 (9.4)	4317 (11.6)	
West Midlands	8184 (10.7)	6042 (16.3)	
East of England	8486 (11.1)	3479 (9.4)	
London	7408 (9.7)	1898 (5.1)	
South East	10 991 (14.4)	5612 (15.1)	
South West	9253 (12.1)	3919 (10.6)	
Unknown or no fixed abode	168 (0.2)	84 (0.2)	
**Ethnic group**			
White	59 746 (78.0)	30 649 (82.6)	<0.001
Mixed	616 (0.8)	206 (0.6)	
Asian or Asian British	2783 (3.6)	983 (2.6)	
Black or black British	3236 (4.2)	641 (1.7)	
Other ethnic group	871 (1.1)	293 (0.8)	
Unknown	9329 (12.2)	4326 (11.7)	
**Comorbidity score**			
<5	72 518 (94.7)	34 690 (93.5)	<0.001
5-10	1936 (2.5)	1126 (3.0)	
11-15	1485 (1.9)	894 (2.4)	
>15	642 (0.8)	388 (1.0)	
**Deprivation score**			
1 (most deprived)	17 663 (23.1)	8008 (21.6)	<0.001
2	16 729 (21.8)	7882 (21.2)	
3	15 528 (20.3)	7711 (20.8)	
4	14 220 (18.6)	7228 (19.5)	
5 (least deprived)	12 308 (16.1)	6209 (16.7)	
Unknown	133 (0.2)	60 (0.2)	
**Year of hysterectomy**			
2004/05	8660 (11.3)	4264 (11.5)	<0.001
2005/06	9001 (11.8)	4246 (11.4)	
2006/07	8372 (10.9)	4087 (11.0)	
2007/08	8409 (11.0)	3952 (10.7)	
2008/09	8025 (10.5)	3775 (10.2)	
2009/10	7819 (10.2)	3740 (10.1)	
2010/11	7646 (10.0)	3531 (9.5)	
2011/12	6767 (8.8)	3262 (8.8)	
2012/13	6021 (7.9)	3157 (8.5)	
2013/14	5861 (7.8)	3084 (8.3)	
**No of previous admissions**			
0	9734 (12.7)	4019 (10.8)	<0.001
1	16 541 (21.6)	7778 (21.0)	
2-10	46 422 (60.6)	23 185 (62.5)	
11- 20	3210 (4.2)	1717 (4.6)	
>20	674 (0.9)	399 (1.1)	
**Hysterectomy code**			
Q074—Total abdominal hysterectomy	49 329 (64.4)	33 095 (89.1)	<0.001
Q078—Other specified abdominal excision of uterus	74 (0.1)	17 (0.1)	
Q079—Unspecified abdominal excision of uterus	606 (0.8)	302 (0.8)	
Q088—Other specified vaginal excision of uterus	240 (0.3)	60 (0.2)	
Q089—Unspecified vaginal excision of uterus	26 332 (34.4)	3624 (9.8)	
**Operation method**			
Laparoscopic	7770 (10.1)	5176 (14.0)	<0.001
Laparoscopic converted to open	537 (0.7)	265 (0.7)	
Non-laparoscopic	68 274 (89.2)	31 657 (85.3)	

The median age of patients was 41 (interquartile range 39-43) years in the ovarian conservation group and 42 (40-44) years in the bilateral removal group. A significant difference existed between the overall demographics of the patients in each group. For example, 16.3% (6042/37 098) of bilateral removal procedures were performed in the West Midlands compared with 10.7% (8184/76 581) of the ovarian conservation operations (P<0.001) (table 1). The number of hysterectomies in the target age group has decreased gradually across the years, from 12 924 in 2004/05 to 8945 in 2013/14 (see supplementary figure).

### Admissions to hospital after index hysterectomy

We found no significant difference in χ^2^ tests between groups in the proportion of patients having an emergency readmission within 30 days (P=0.85) or 90 days (P=0.47) (table 2). Some individual differences in reason for readmission were statistically significant (table 2), but the magnitude of difference was small in each case (never exceeding 0.4%), and the direction of effect was inconsistent—for example, an increase in haemorrhage with ovarian conservation and of infection with bilateral removal.

**Table 2 tbl2:** 30 and 90 day post-hysterectomy admissions by group and by reason for readmission. Values are numbers (percentages) unless stated otherwise

Readmissions	30 day readmission				90 day readmission			
	Ovarian conservation group (n=76 581)	Bilateral removal group (n=37 098)	P value		Ovarian conservation group (n=76 581)	Bilateral removal group (n=37 098)	P value	
Total (% of readmissions)	6671 (8.7)	3219 (8.7)	0.85		8081 (10.6)	3967 (10.7)		0.47
T810—Haemorrhage resulting from a procedure	1531 (2.0)	606 (1.6)	<0.001		1590 (2.1)	632 (1.7)		<0.001
T814—Infection following a procedure	1064 (1.4)	593 (1.6)	0.006		1140 (1.5)	610 (1.6)		0.04
R104—Other and unspecified abdominal pain	449 (0.6)	243 (0.7)	0.16		573 (0.7)	337 (0.9)		0.004
R103—Pain localised to other parts of abdomen	311 (0.4)	141 (0.4)	0.51		427 (0.6)	204 (0.5)		0.87
N390—Other disorders of urinary system	273 (0.4)	128 (0.3)	0.76		321 (0.4)	144 (0.4)		0.44
T818—Other complications of procedures, not elsewhere classified	240 (0.3)	126 (0.3)	0.46		285 (0.4)	141 (0.4)		0.84
T813—Disruption of operation wound, not elsewhere classified	173 (0.2)	109 (0.3)	0.03		188 (0.2)	117 (0.3)		0.03
K590—Constipation	213 (0.3)	107 (0.3)	0.76		246 (0.3)	131 (0.4)		0.38
N939—Abnormal uterine and vaginal bleeding, unspecified	233 (0.3)	96 (0.3)	0.18		270 (0.4)	108 (0.3)		0.09
R074—Chest pain	95 (0.1)	45 (0.1)	0.90		132 (0.2)	68 (0.2)		0.68
N898—Other specified non-inflammatory disorders of vagina	107 (0.1)	44 (0.1)	0.36		135 (0.2)	55 (0.1)		0.28

The rate of admission for ischaemic heart disease was 1.60% (1227/76 581) in the ovarian conservation group compared with 2.02% (751/37 098) in the bilateral removal group. This gives an absolute rate difference of 0.42% (adjusted hazard ratio 0.85, 95% confidence interval 0.77 to 0.93; P<0.001) (fig 1). The median time to event was 56 (interquartile range 25-85) months for the ovarian conservation group patients and 51 (23-81.5) months for the bilateral removal group (P<0.001).

**Figure f1:**
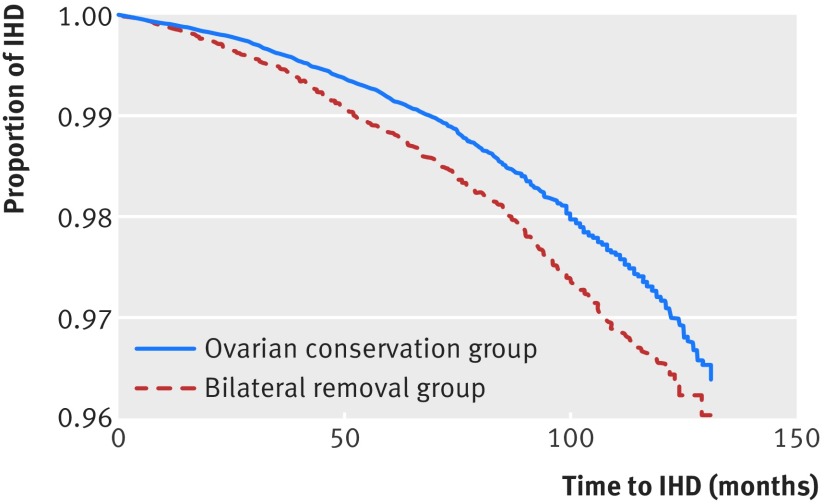
**Fig 1** Kaplan-Meier survival curve for time (in months) from hysterectomy to admission for ischaemic heart disease by type of ovary removal, for all patients at risk

The rate of admission with any cancer diagnosis was also lower in the ovarian conservation group (2.80%) than in the bilateral removal group (3.49%) (P<0.001), with an absolute rate difference of 0.69% (adjusted hazard ratio 0.83, 0.78 to 0.89; P<0.001). Table 3 gives results for individual cancers. We saw an immediate increase in the finding of ovarian cancer when both ovaries were removed (0.29% *v* 0.07%; P<0.001), with an absolute rate difference of 0.22%, but the incidence converged over follow-up (fig 2). The median time to ovarian cancer diagnosis was 4 (interquartile range 4-14.75) months in the bilateral removal group compared with 54 (28.75-99) months in the ovarian conservation group (P<0.001). We return to this point in the discussion.

**Table 3 tbl3:** Post-hysterectomy admissions for patients with cancer diagnosis. Values are numbers (percentages) unless stated otherwise

Cancer diagnosis	Ovarian conservation (n=76 581)	Bilateral removal (n=37 098)	P value (univariate)	Adjusted HR^*^ (95% CI)	P value (multivariate)	Favours
Any cancer	2141 (2.80)	1296 (3.49)	<0.001	0.83 (0.78 to 0.89)	<0.001	Ovarian conservation
Breast cancer (C50)	784 (1.02)	361 (0.97)	0.42	1.34 (1.15 to 1.55)	<0.001	Bilateral removal
Ovarian cancer (C56)	56 (0.07)	108 (0.29)	<0.001	0.26 (0.19 to 0.37)	<0.001	(See text)
Other reproductive cancer (C51, C52, C53, C54, C55, C57, C58)	69 (0.09)	45 (0.12)	0.12	0.75 (0.52 to 1.10)	0.14	Neither
Lung cancer (C34)	90 (0.12)	69 (0.19)	0.004	0.66 (0.48 to 0.91)	0.01	Ovarian conservation
Colon cancer (C18)	81 (0.11)	88 (0.24)	<0.001	0.50 (0.37 to 0.68)	<0.001	Ovarian conservation
Bladder cancer (C67)	49 (0.06)	44 (0.12)	0.003	0.60 (0.40 to 0.90)	0.01	Ovarian conservation
Other cancer	1475 (1.93)	906 (2.44)	<0.001	0.84 (0.77 to 0.91)	<0.001	Ovarian conservation

Total number of patients with “any cancer” does not equal sum of individual cancers, as some patients had more than one type of cancer.

*Hazard ratios less than 1 favour ovarian conservation.

**Figure f2:**
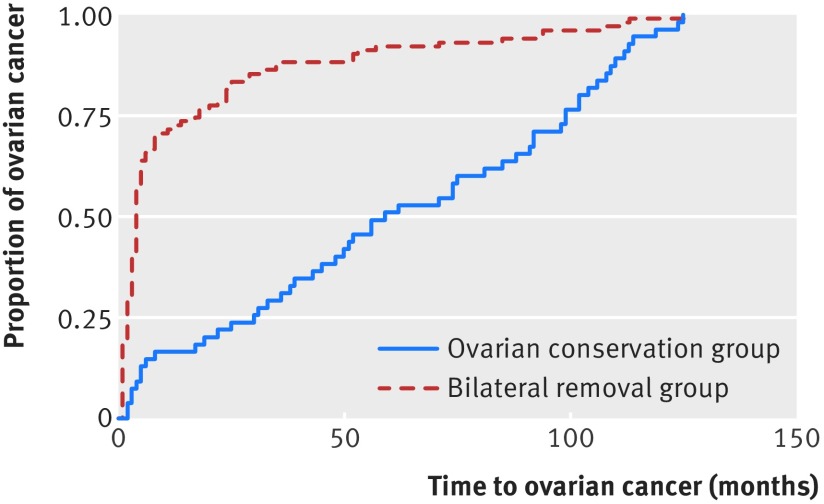
**Fig 2** Kaplan-Meier survival curve for time to post-hysterectomy ovarian cancer by type of ovary removal

Overall, 1.01% of patients (1145/113 679) had a diagnosis of breast cancer after their hysterectomy. The rate was slightly higher in the ovarian conservation group: 1.02% (784/76 581) versus 0.97% (361/37 098) in the bilateral removal group (adjusted hazard ratio 1.34, 1.15 to 1.55; P<0.001). The median time to event was 57.5 months for the ovarian conservation group and 51 months for the bilateral removal group (P=0.02). Admission rates were significantly lower in the ovarian conservation group for cancers of the lung and bladder and highly significantly lower (P<0.001) for colon cancer and “any other” cancer (table 3).

The rate of admission for attempted suicide was similar in the two groups: 2.13% (1632/76 581) in the ovarian conservation group and 2.07% (768/37 098) in the bilateral removal group, yielding an absolute rate difference of 0.05 (adjusted hazard ratio 1.02, 0.94 to 1.11; P=0.61). The median time to event was 39 (interquartile range 19-65) months for patients in the ovarian conservation group compared with 36 (16-63) months in the bilateral removal group (P=0.09).

### Death rates: HES linkage to ONS

The rate of all cause death was lower in the group in which ovarian tissue was conserved (0.60% *v* 1.01%; P<0.001), with an absolute rate difference of 0.41% (adjusted hazard ratio 0.64, 0.55 to 0.73; P<0.001). In the ovarian conservation group, 13.4% (61/456) of deaths occurred within the first 12 months of hysterectomy compared with 17.3% (65/376) of the bilateral removal group (P=0.12) (fig 3). If ovarian cancer deaths are excluded from the above analysis (see discussion for rationale), the association is more extreme in favour of ovarian conservation (hazard ratio 0.60, 0.52 to 0.69; P<0.01).

**Figure f3:**
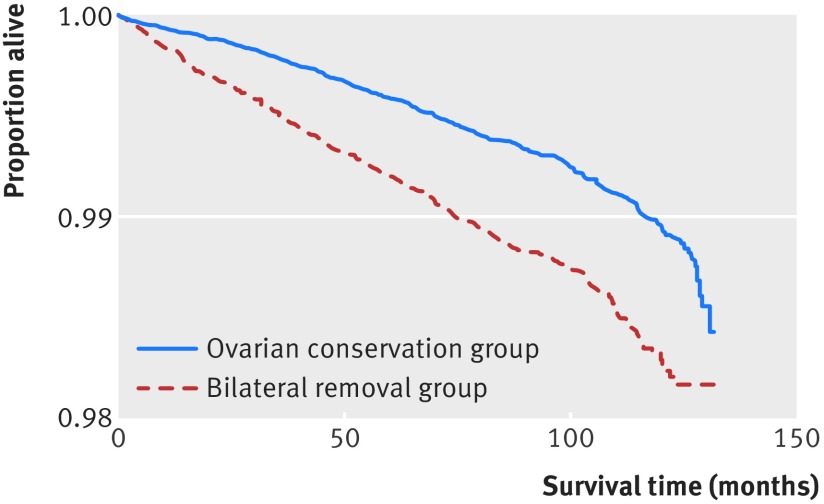
**Fig 3** Kaplan-Meier survival curve for time (in months) from hysterectomy by type of ovary removal (all deaths)

Table 4 gives the causes of death in the two groups. A total of 832 deaths were observed over the study, 46 from heart disease and 472 from cancer, representing 0.04% and 0.42% of all cases respectively.

**Table 4 tbl4:** Deaths by cause in ovarian conservation and bilateral removal groups during follow-up

Cause of death	Total (% of all cases)		P value (χ^2^ tests)	Adjusted HR^*^ (95% CI)	P value (multivariate)	Favours
	Ovarian conservation (n=76 581)	Bilateral removal (n=37 098)				
All deaths	456 (0.60)	376 (1.01)	<0.001	0.64 (0.55 to 0.73)	<0.001	Ovarian conservation
Heart disease	23 (0.03)	23 (0.06)	0.01	0.50 (0.28 to 0.90)	0.02	Ovarian conservation
Any cancer	237 (0.31)	235 (0.63)	<0.001	0.54 (0.45 to 0.65)	<0.001	Ovarian conservation
Breast cancer (C50)	46 (0.06)	38 (0.10)	0.01	0.61 (0.39 to 0.94)	0.03	Ovarian conservation
Ovarian cancer (C56)	7 (0.01)	18 (0.05)	<0.001	0.21 (0.09 to 0.50)	<0.001	Ovarian conservation
Reproductive cancer (C51, C52, C53, C54, C55, C57, C58)	7 (0.01)	0 (0)	0.51	–	–	Neither
Lung cancer (C34)	45 (0.06)	24 (0.06)	0.70	0.95 (0.58 to 1.57)	0.85	Neither
Colon cancer (C18)	18 (0.02)	22 (0.06)	0.003	0.47 (0.25 to 0.88)	0.02	Ovarian conservation
Bladder cancer (C67)	13 (0.02)	26 (0.07)	<0.001	0.60 (0.40 to 0.91)	0.02	Ovarian conservation
Other cancer	109 (0.14)	107 (0.29)	<0.001	0.53 (0.40 to 0.69)	<0.001	Ovarian conservation

Total number of patients with “any cancer” does not equal sum of individual cancers, as some patients had more than one type of cancer.

*Hazard ratios less than 1 favour ovarian conservation.

The crude rate of death from heart disease was 0.03% (23/76 581) in the ovarian conservation group compared with 0.06% (23/37 098) in the bilateral removal group (adjusted hazard ratio 0.50, 0.28 to 0.90; P=0.02). The rate of death from cancer was also lower in the ovarian conservation group (0.31%; 237/76 581) than in the bilateral removal group (0.63%; 235/37 098), with a difference of 0.32% (adjusted hazard ratio 0.54, 0.45 to 0.65; P<0.001). As can be seen in table 4, deaths were significantly less common in the ovarian conservation group than in the bilateral removal group in each individual category of cancers, except the very rare cases of “reproductive cancers other than ovarian and breast” and lung cancer. We found no absolute rate difference for completed suicide between patients in the ovarian conservation group and the bilateral removal group (adjusted hazard ratio 1.03, 0.39 to 2.72; P=0.95).

We also tested the main outcomes (all cause death, ischaemic heart disease death, cancer death, suicide death, ischaemic heart disease admission, cancer admission, and suicide admission) by using Cox regression, after propensity score matching, but the results tell the same story as our initial findings (table 5).

**Table 5 tbl5:** Cox regression after propensity score matching, adjusted for age, deprivation, Charlson comorbidity score, and number of admissions before hysterectomy and standard Cox regression

Outcomes	After propensity matching			After Cox regression	
	Adjusted hazard ratio^*^ (95% CI)	P value		Adjusted hazard ratio^*^ (95% CI)	P value
Post-hysterectomy IHD admission	0.84 (0.76 to 0.92)	<0.001		0.85 (0.77 to 0.93)	0.001
Post-hysterectomy cancer admission	0.84 (0.78 to 0.90)	<0.001		0.83 (0.78 to 0.89)	<0.001
Post-hysterectomy suicide admission	1.06 (0.96 to 1.78)	0.26		1.02 (0.94 to 1.11)	0.61
All cause death	0.78 (0.66 to 0.92)	0.003		0.64 (0.55 to 0.73)	<0.001
Heart disease death	0.50 (0.28 to 0.90)	0.02		0.50 (0.28 to 0.90)	0.02
Cancer death	0.61 (0.50 to 0.74)	<0.001		0.54 (0.45 to 0.65)	<0.001
Suicide death	1.03 (0.40 to 2.73)	0.95		1.03 (0.39 to 2.72)	0.95

IHD=ischaemic heart disease.

*Hazard ratios less than 1 favour ovarian conservation.

## Discussion

All cause mortality was lower when ovarian tissue was conserved than when all ovarian tissue was removed, with a statistically significant difference of 0.41 percentage points. This amounts to one death in about 240 operations over 10 years, which is clinically significant. The survival curves diverge from the first year, and the result is highly significant, even though the mortality rate was less than 2% overall within the 10 year time frame of this large study. We found a lower rate of ischaemic heart disease events in the ovarian conservation group. Again, the time to event curves diverge from the first postoperative year. Cancer deaths were also reduced overall. The overall rate of suicide (attempted or completed) was high (nearly 2%) but did not differ by oophorectomy status, and this is in line with a previous study on this point.^6^

Our finding in these and other respects are generally consistent internally (the admission data corroborate the mortality data) and externally (the mortality data in this study corroborate findings in the literature). We now discuss the question of internal and external consistency.

### Internal consistency

The headline finding of a reduction in admissions for ischaemic heart disease is consistent with lower death rates from heart disease. Death from heart disease is a less common endpoint than overall death, and confidence limits are thus wider are but still “significant” at P=0.01. Likewise, overall cancer data are consistent with lower rates of admission being mirrored by lower death rates across cancer as a whole. The results are consistent with respect to colon cancer, bladder cancer, and “other cancer” (risk of all reduced after ovarian conservation), whereas cancer of the lung showed reduced admissions with ovarian conservation but no difference in death rates. The anomaly is breast cancer, for which adjusted admission rates were higher with ovarian conservation but death rates were lower. We return to this point.

### External consistency

The main study with which we draw comparison is the large Nurses’ Health Study, which is not subject to publication bias,^10^ is large, and has long follow-up. Here we argue that our results are consistent where this would be expected and that, where they diverge, this can be explained by the main limitation of our study—limited duration of follow-up. Our results are entirely consistent with respect to overall mortality and heart disease death rates. They are also consistent with respect to cancer as a whole, and with respect to certain individual cancers, such as colon cancer. The literature as a whole (table 6) shows a reduction in colon cancer (incidence and mortality) with ovarian conservation, which is replicated in this study. The situation regarding cancer of the lung is more ambiguous in the literature, and this is reflected in the null result for mortality observed in this study. Our results diverge from both the Nurses’ Health Study and other literature with respect to ovarian cancer admissions and breast cancer. We now discuss these two cancers.

**Table 6 tbl6:** Effects of ovarian conservation or natural menopause versus bilateral ovarian removal on incidence of cancer in literature

Cancer type	Reference^*^	Study design	Sample size	Outcome	Comparison	Favours	Result (95% CI)
Ovarian	Parker et al 2009^1^	Prospective, observational	29 380	Incidence	Bilateral *v* ovarian conservation	Bilateral	HR 0.04 (0.01 to 0.09)^†^
	Parker et al 2013^3^	Prospective, observational	30 117	Mortality	Bilateral *v* ovarian conservation	Bilateral	HR 0.06 (0.02 to 0.17)^‡^
Breast	Parker et al 2009^1^	Prospective, observational	29 380	Incidence	Bilateral *v* ovarian conservation	Bilateral	HR 0.75 (0.68 to 0.84)^‡^
	Parker et al 2013^3^	Prospective, observational	30 117	Mortality	Bilateral *v* ovarian conservation	No significant difference	HR 0.89 (0.69 to 1.15)^‡^
Breast (oestrogen receptor positive)	Boggs et al 2014^11^	Prospective, observational	44 514	Incidence	Bilateral *v* ovarian conservation	Bilateral	HR 0.62 (0.45 to 0.85)^‡^
Breast (oestrogen receptor negative)	Boggs et al 2014^11^	Prospective, observational	44 514	Incidence	Bilateral *v* ovarian conservation	No significant difference	HR 1.03 (0.67 to 1.59)^‡^
Lung	Parker et al 2009^1^	Prospective, observational	29 380	Incidence	Bilateral *v* ovarian conservation	Ovarian conservation	HR 1.26 (1.02 to 1.56)^‡^
	Parker et al 2013^3^	Prospective, observational	30 117	Mortality	Bilateral *v* ovarian conservation	Ovarian conservation	HR 1.29 (1.04 to 1.61)^‡^
	Koushik et al 2009^12^	Case-control	999	Incidence	Bilateral *v*. natural menopause	Bilateral	OR 1.90 (1.18 to 3.06)^‡^
	Boggs et al 2014^11^	Prospective, observational	44 514	Incidence	Bilateral *v* ovarian conservation	No significant difference	HR 0.97 (0.65 to 1.45)^‡^
	Pesatori et al 2013^13^	Case-control	906	Incidence	Bilateral *v* natural menopause	No significant difference	OR 1.03 (0.62 to 1.71)^‡^
	Baik et al 2009^14^	Prospective, observational	1729	Incidence	Bilateral *v* natural menopause	No significant difference	OR 1.06 (0.90 to 1.25)^‡^
	Gallagher et al 2014^15^	Prospective, observational	3637	Incidence	Bilateral *v* natural menopause	No significant difference	HR 1.39 (0.96 to 2.00)
Adenocarcinomas of lung	Koushik et al 2009^12^	Case-control	999	Incidence	Ovarian conservation *v* natural menopause	No significant difference	OR 1.23 (0.59 to 2.56)^‡^
	Koushik et al 2009^12^	Case-control	999	Incidence	Bilateral *v* natural menopause	Bilateral	OR 1.79 (1.03 to 3.10)^‡^
Colorectal	Parker et al 2009^1^	Prospective, observational	29 380	Incidence	Bilateral *v* ovarian conservation	No significant difference	HR 1.23 (0.98 to 1.54)^‡^
	Parker et al 2013^3^	Prospective, observational	30 117	Mortality	Bilateral *v* ovarian conservation	Ovarian conservation	HR 1.49 (1.02 to 2.18)^‡^
	Segelman et al 2016^16^	Prospective, observational	195 973	Incidence	Bilateral *v* ovarian conservation	Ovarian conservation	HR 2.28 (1.33 to 3.91)^‡^
	Boggs et al 2014^11^	Prospective, observational	44 514	Incidence	Bilateral *v* ovarian conservation	No significant difference	HR 1.31 (0.85 to 2.00)^‡^
All cancers	Parker et al 2009^1^	Prospective, observational	29 380	Incidence	Bilateral *v* ovarian conservation	Bilateral	HR 0.92 (0.86 to 0.98)^†^
	Parker et al 2013^3^	Prospective, observational	30 117	Mortality	Bilateral *v* ovarian conservation	Ovarian conservation	HR 1.13 (1.06 to 1.21)^‡^

HR=hazard ratio; OR=odds ratio.

*Parker et al (2009, 2013) refer to Nurses’ Health Study.

†Age adjusted.

‡Multivariable adjusted.

### Ovarian cancer

Readmission rates for ovarian cancer were higher when both ovaries were removed. The excess of cancers appears within a few weeks after ovarian removal, and this would not seem to be compatible with a biological causation. We believe that this initially paradoxical finding has a simple explanation. When the surgeon encounters a previously unsuspected thick walled cyst that is not obviously cancerous, the possibilities are a benign neoplasm (such as a serous cystadenoma), a “borderline malignancy,” or a cancer that has not broken through the ovarian capsule (stage 1A). In such a scenario, the surgeon will usually remove the ovary. There is a tendency for neoplastic cysts to be bilateral, and in some patients the affected ovary will be their only remaining ovary. The HES record, which does not include outpatient attendances, will be updated when the patient is readmitted for further treatment. Further evidence for this explanation can be found in convergence of the curves in the two groups during follow-up (fig 2). Simple extrapolation of these curves yields a result compatible with the Nurses’ Health Study and with the theoretical expectation that removal of the ovaries (along with the proximal end of the fallopian tubes) substantially reduces the risk of ovarian cancer (which includes some cases that originated from the proximal fallopian tubes).^17^

### Breast cancer

Our results agree with the Nurses’ Health Study with respect to breast cancer—they show an increased incidence of breast cancer with ovarian conservation, and we corroborate this by finding an increased rate of post-hysterectomy admission with ovarian conservation. Likewise, both studies found lower rates of death from breast cancer with ovarian conservation—significant in this study, non-significant in the Nurses’ Health Study. It is possible that, with longer follow-up, our study will show that death rates from breast cancer eventually rise in the ovarian conservation group, in line with higher admission rates in this group. However, cancers associated with hormone replacement therapy tend to be less advanced clinically than those among women who have not used such therapy.^18^ So incidence may not be mirrored in death rates when hormones are manipulated in this cancer.

### Theoretical implications

Our results are broadly in line with theory. In particular, theoretical reasons exist to suspect that a drop in endogenous oestrogen concentrations may increase the risk of cardiovascular disease in iatrogenic menopause, just as it seems to do in non-iatrogenic premature menopause.^19^ ^20^ The increased risk of breast cancer associated with oestrogen may be reflected in higher incidence of breast cancer in the ovarian conservation group. The risk of colon cancer is reduced by hormone replacement therapy in both randomised controlled trials and other types of study,^21^ ^22^ and this is consistent with a lower incidence of this cancer in the ovarian conservation group in our study and in others. There are sound reasons for thinking that oophorectomy will protect against ovarian cancer (even if a proportion of the latter arise in the proximal end of the fallopian tube), and this is consistent with the trends depicted in figure 2.

### Strengths of this study

Our study was large, including 113 679 participants—33% in the bilateral group and 67% in the ovarian conservation group. As far as we know, it is the largest study to examine this question and included a whole country. A caveat with large studies is that a bias of a given magnitude is more likely to lead to a false positive result when studies are large and observations precise than when observations are less precise. However, database studies may have greater external validity than prospective non-randomised cohort studies in which people who decline to participate may vary systematically from those who agree.^23^ People with previous heart disease may be systematically less likely to have bilateral removal than people with no such history. However, we found no sign of this in the data (table 1); if anything, the ovarian conservation group had more comorbidities and less deprivation than the comparator bilateral oophorectomy group.

### Limitations of this study

The duration of follow-up was limited to a maximum of 10 years, but statistical trends emerged despite this, in part because the study was large. Limitations in earlier versions of the HES data precluded longer follow-up. We plan to re-examine the cohort at a later date to examine trends over the long term. It is interesting that differences in ischaemic heart disease appeared so rapidly after the operation. The effect regarding breast cancer may reverse during longer follow-up, for reasons given above.

The data available in the database are not as detailed as might be achieved in a prospective study. In particular, we do not have information on use of hormone replacement therapy. However, our results represent the pragmatic association between surgical type and outcome, irrespective of whether this resulted from failure to start hormone replacement therapy, failure to maintain its use, or a combination of factors. We also do not have data with respect to quality of life in general and acute oestrogen deficiency in particular, but such evidence is available from other, more in-depth, studies.^6^

The study was not randomised, and adjustment may have omitted factors that could have affected selection of operation type. This is exactly what seems to have happened in the particular case of ovarian cancer. That, however, is a special case as the decision to remove the ovaries arises during the operation itself, as described above. We deliberately selected patients in the age range of 35 to 45, to isolate the age group in which the trade-off between removal and conservation of ovarian tissue yields the greatest decision uncertainty. Below this age range, the case for removing all ovarian tissue is weak in the absence of a specific risk factor. Below the age of 35, the relatively low incidence of hysterectomy (4.2% of hysterectomies in the HES database), of oophorectomy given hysterectomy (25% in the HES database), and of a clinical event during follow-up, make this an unpromising age range for study, pending longer-term follow-up. Beyond the age of 45, the woman enters the peri-menopausal period when arguments for ovarian conservation are less compelling, and the Nurses’ Health Study did not find a protective (or harmful) effect from ovarian removal in this group of women. There may be some, yet to be discovered, effect of ovarian removal/conservation in this older age group, and we plan to investigate this possibility when more women/years of “exposure” have been accrued.

### Clinical relevance

Our data corroborate theory and the Nurses’ Health Study data in finding an association between bilateral oophorectomy and ischaemic heart disease. Cancer deaths and overall mortality were also increased, again in line with the Nurses’ Health Study. Although selection bias remains a possibility, the corroboration between two different methods may offer a measure of confirmation beyond that of just one more replication of a similar study.^24^ The overall incidence of hysterectomy is declining, in contradiction of an earlier prediction from one of the authors.^25^ However, the data show only a slow decline in use of this operation; nearly 9000 women had hysterectomy for a benign condition in the target age range in 2014. These women are likely to be interested in, and may be influenced by, our study. Forty per cent of women with no specific risk factors for reproductive cancer had their ovaries removed during abdominal hysterectomy in the 35-45 age group. This might be a higher proportion than would be expected among women who were fully cognisant of the worse health outcomes with bilateral removal reported here. In that case, we might expect the proportion of women who select bilateral ovarian removal to decline as the health risks that must be traded for a reduced incidence of ovarian cancer come into sharper focus.

**What is already known on this topic**

Many pre-menopausal women with no specific indication have both ovaries removed during hysterectomy as a prophylactic measure against the risk of ovarian cancer
Removal of the ovary may have long term harmful effects, which must be offset against the benefit conferred by protection from ovarian cancer
A decrease in endogenous oestrogen may increase the hazard of cardiovascular disease or all cause mortality, but little empirical evidence for this exists

**What this study adds**

Patients who had at least one ovary conserved had a significantly lower rate of all cause mortality than patients who had both ovaries removed
Reduced admissions for ischaemic heart disease and cancer were mirrored in lower deaths from heart disease and cancer in patients who had ovarian conservation rather than bilateral ovarian removal

## Web Extra

Extra material supplied by the author

## Notes

Ethical approval: As only pseudonymised information was used in this study, ethics approval was not needed. HES data are available to University Hospitals Birmingham under a data sharing agreement for the purposes of service evaluation.

Data sharing: No additional data available.

Transparency: The lead author (the manuscript’s guarantor) affirms that this manuscript is an honest, accurate, and transparent account of the study being reported; that no important aspects of the study have been omitted; and that any discrepancies from the study as planned have been explained.
