# Supplementary material for: Removal of all ovarian tissue versus conserving ovarian tissue at time of hysterectomy in premenopausal patients with benign disease: study using routine data and data linkage
Source: BMJ. 2017 Feb 6;356:j372. doi: 10.1136/bmj.j372 (PMC5421461; doi:10.1136/bmj.j372)

SUPPLEMENTARY FIGURE

Trends in hysterectomy ages 35-45 over the study period, overall and by operation type.

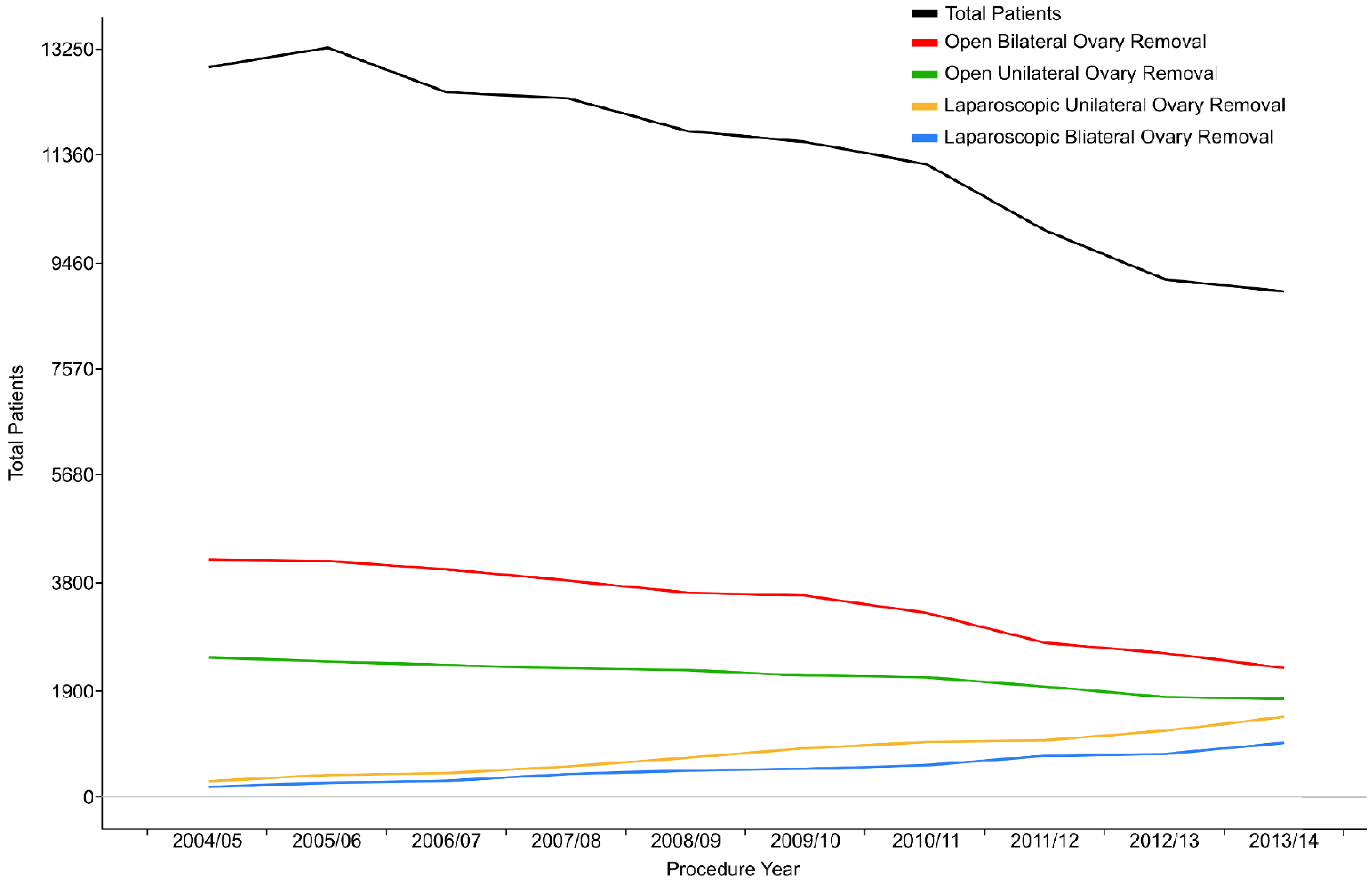

Supplement: Supplementary file 1 — Supplementary figure [file mytj034990.ww1.pdf]
